# Abundance of BER-related proteins depends on cell proliferation status and the presence of DNA polymerase β

**DOI:** 10.1093/jrr/rrv010

**Published:** 2015-03-31

**Authors:** Mizuki Yamamoto, Ryohei Yamamoto, Shigeo Takenaka, Satoshi Matsuyama, Kihei Kubo

**Affiliations:** 1 Department of Advanced Pathobiology, Graduate School of Life and Environmental Sciences, Osaka Prefecture University, 1–58 Rinku Ourai-Kita, Izumisano, Osaka 598–8531, Japan; 2 Department of Structural and Functional Biosciences for Animals, Graduate School of Life and Environmental Sciences, Osaka Prefecture University, 1–58 Rinku Ourai-Kita, Izumisano, Osaka 598–8531, Japan

**Keywords:** BER, MPG, mouse, cell proliferation, POL β

## Abstract

In mammalian cells, murine *N*-methylpurine DNA glycosylase (MPG) removes bases damaged spontaneously or by chemical agents through the process called base excision repair (BER). In this study, we investigated the influence of POL β deficiency on MPG-initiated BER efficiency and the expression levels of BER-related proteins in log-phase and growth-arrested (G_0_) mouse embryonic fibroblasts (MEFs). G_0_ wild-type (WT) or POL β–deficient (*Pol β–KO*) cells showed greater resistance to methyl methanesulfonate than did log-phase cells, and repair of methylated bases was less efficient in the G_0_ cells. *Apex1* mRNA expression was significantly lower in *Pol β*–KO or G_0_ WT MEFs than in log-phase WT MEFs. Moreover, although *Mpg* mRNA levels did not differ significantly among cell types, MPG protein levels were significantly higher in log-phase WT cells than in log-phase *Pol β–*KO cells or either type of G_0_ cells. Additionally, proliferating cell nuclear antigen protein levels were also reduced in log-phase *Pol β–*KO cells or either type of G_0_ cells. These results indicated that MPG-initiated BER functions mainly in proliferating cells, but less so in G_0_ cells, and that POL β may be involved in regulation of the amount of intracellular repair proteins.

## INTRODUCTION

In mammalian cells, DNA is damaged by various endogenous and environmental factors [1, 2]. Under physiological conditions, base damage is the most common DNA lesion and is estimated to occur spontaneously in a single proliferating mammalian cell about 10 000 times each day [3]. This damage can cause cell death and mutation via induction of stalled replication forks and base mispairing. The base excision repair (BER) mechanism is highly conserved from prokaryotes to mammalian cells, but the intricate relationships among various repair proteins have hindered complete documentation of the BER mechanism. Also, the amount of spontaneous base damage has been estimated for ‘proliferating’ cells, but information on this amount in non-proliferative cells is relatively limited.

Sykora and colleagues recently showed that differentiated human neural cells exhibit resistance to methyl methanesulfonate (MMS) and that MMS-induced DNA damage is repaired slightly less efficiently in terminally differentiated cells than in differentiating cells [4]. MMS has been used to induce base damage in many studies to investigate BER mechanisms because the product spectrum is well established, with >90% of the products being the toxic 3-methyladenine (3meA, 11%) or the less harmful 7-methylguanine (7meG, 83%) [5]. In our previous study, the yield of methylated bases induced by MMS treatment in log-phase HeLa cells is estimated to be 7–8/10^5^ nucleotides/mM [6]. Even in rapidly growing cells, repair of methylated bases is slow, and about half of the MMS-induced damage is unrepaired 24 h after treatment [7]. In rat liver, the most abundant product, 7meG, has a half-life of about 48 h in non-proliferative cells [8], and accumulated damage increases with rat age [9]. The differences in removal rates between these cells may be ascribed to their growth states.

Most bases that are methylated by MMS are exclusively removed by *N*-methylpurine DNA glycosylase (MPG), which initiates single-nucleotide BER (SN-BER). MPG has a broad substrate specificity, including 3meA, 7meG, hypoxanthine (Hx), xanthine and ethenoadenine [10–13]. In cells overexpressing MPG, the number of BER intermediates, such as AP sites and single-strand breaks, increases after treatment with alkylating agents as a result of imbalanced BER, and these BER intermediates can lead to chromosomal aberrations and sister chromatid exchange [14, 15]. These results may be attributable to uncontrolled MPG-mediated conversion of 7meG into more toxic BER intermediates [16]. In contrast, MPG-deficient HeLa cells display hypersensitivity to alkylating agents, and MPG-deficient mouse ES cells have higher levels of chromosome damage than do WT cells [17, 18]. Taken together, these results show that control of MPG expression level during BER is critical for genetic integrity.

Because proteins involved in long-patch BER (LP-BER), such as proliferating cell nuclear antigen (PCNA), flap endonuclease 1 (FEN1), and DNA ligase I, are downregulated in non-dividing cells [4], the SN-BER pathway, in which POL β plays a pivotal role, is likely to be the predominant process responsible for repair of alkylated base lesions. Therefore, POL β deficiency should result in elevated sensitivity to MMS in non-proliferative cells, but less so in log-phase cells. During SN-BER, AP endonuclease 1 (APEX1) cleaves the DNA strand at the 5′-side of an AP site and leaves a 5′-deoxyribose phosphate (5′-dRP). POL β removes the 5′-dRP using its lyase activity and inserts a correct nucleotide. Exponentially growing POL β–deficient mouse embryonic fibroblasts (MEFs) are hypersensitive to MMS [19, 20], and expression of just the POL β 5′-dRP lyase domain in these cells abrogates this hypersensitivity [21]. The cytotoxicity of 5′-dRP created by MPG and APEX1 may result in the hypersensitive phenotype of POL β–deficient cells. Moreover, hypersensitivity to MMS or *N*-methyl-*N*'-nitro-*N*-nitrosoguanidine (MNNG) does not occur in cells deficient for both POL β and MPG [22]. These results indicate that hypersensitivity of POL β–deficient cells to MMS depends on MPG. Using a comet assay, Pascucci *et al*. showed that POL β deficiency decreases the efficiency of SSB repair more significantly in G_1_ phase MEFs than in S phase MEFs [23].

These results may indicate that BER-related proteins are downregulated by a lack of POL β, because the activities of these proteins are thought to be strictly controlled. However, more direct evidence is needed to convincingly document these relationships. In this study, we investigated the mRNA and protein levels from BER-related genes in log-phase and non-proliferating (G_0_) mouse cells to understand the contribution of the BER system to cell viability.

## MATERIALS AND METHODS

### Antibodies

Anti-MPG antibody (Proteintech, Chicago, IL, USA), anti-XRCC1 and anti-APEX1 antibodies (Abcam, Cambridge, England), anti-POL β antibody (Trevigen, Gaithersburg, MD, USA), anti-β-Actin antibody (Sigma-Aldrich, St Louis, MO, USA) and anti-PCNA antibody (Cell Signaling Technology, Danvers, MA, USA) were used in the study.

### Cell culture and MMS treatment

Mβ16tsA and Mβ19tsA MEFs, which were kindly provided by Dr Masahiko Miura (Tokyo Medical and Dental University), were used as wild-type (WT) and POL β–deficient (*Pol β–*KO) cells, respectively [19, 20]. Cells were cultured in Eagle's MEM (Nissui Pharmaceutical Co. Ltd, Tokyo, Japan) containing 10% fetal bovine serum (FBS; Thermo Scientific, Waltham, MA, USA), 1% sodium pyruvate, and MEM non-essential amino acids (Life Technologies, Carlsbad, CA, USA) at 37°C in a humidified atmosphere containing 5% CO_2_. To generate cultures with cells in G_0_ phase, 80% confluent cultures were incubated in FBS-free Eagle's MEM for 24 h. Cell cycle distribution was assessed with propidium iodide (PI) and FACSCalibur^TM^ (***Becton, Dickinson,*** Franklin Lakes, NJ, USA). Log-phase and G_0_ cells were incubated in PBS (137 mM NaCl, 2.7 mM KCl, 4.3 mM Na_2_HPO_4_, 1.4 mM KH_2_PO_4_, 0.9 mM CaCl_2_, 0.49 mM MgCl_2_) containing MMS (Sigma–Aldrich) at 37°C for 1 h. After MMS treatment, cells were immediately washed twice with Hank's balanced salt solution (HBSS; Sigma–Aldrich). For each ARP assay, DNA was extracted immediately and 24 h after MMS treatment.

### ARP assay

Each ARP assay was performed as described previously with slight modifications [6]. Cells were harvested by trypsinization, and DNA was extracted via the neutral guanidine thiocyanate-phenol-chloroform method [24, 25]. The prepared DNA was treated with 5 mM aldehyde-reactive probe (ARP; Dojindo Molecular Technologies, Kumamoto, Japan) in TE buffer (10 mM Tris-HCl, pH 8.0, 1 mM EDTA) at 37°C for 1 h. DNA recovered by ethanol precipitation was dissolved in TE buffer, and the DNA concentration was adjusted to 1 μg/ml. DNA solution (200 μl) was added to each well of a protamine-coated plate and incubated at 37°C for 1 h. After washing the plate with TPBS (PBS(-) containing 1% Tween 20), 100 μl of 1:500 diluted ABC solution (avidin-biotinylated horseradish peroxidase complex; Vectastain ABC kit, Vector Laboratories, Burlingame, CA, USA) was added, and the mixture was incubated at room temperature for 30 min. After washing with TPBS, 200 μl of HRP substrate solution (0.2 M Na_2_HPO_4_, 0.1 M citric acid, 0.7 mg/ml *o*-phenylenediamine, 0.5% H_2_O_2_) was added into each well and mixtures were incubated at room temperature for 30 min in the dark. Each reaction was stopped by adding 40 μl of 8 N H_2_SO_4,_ and the absorbance in each well was measured at 495 nm with a Model 680 Microplate Reader (Bio-Rad, Irvine, CA, USA).

### Depurination of methylpurines

In order to quantify the total amount of methylpurines, DNA was dissolved in neutral depurination buffer (0.1 M NaCl, 10 mM citric acid, 10 mM K_2_HPO_4_, pH7.5) and incubated at 80°C for 16 h to convert methylated bases to AP sites [6]. The depurinated DNA was treated with 5 mM ARP for the ARP assays described above.

### Preparation of standard DNA containing AP sites

Preparation of standard DNA containing AP sites was performed according to Lindahl and Nyberg's procedure [3]. Calf thymus DNA (Sigma–Aldrich) was dissolved in TE buffer and purified by phenol-chloroform extraction and ethanol precipitation. The purified DNA was treated with 100 μg/ml RNase A (Sigma) at 37°C for 1 h and then subject to phenol–chloroform extraction and ethanol precipitation. The DNA solution was dialyzed against AP buffer (10 mM sodium citrate, 100 mM NaCl, pH 5.0) and diluted at 1 mg/ml; dialyzed DNA was incubated at 70°C for 0–50 min and recovered by ethanol precipitation; this procedure is reported to form 1 AP site/10^4^ nucleotides/9.26 min [3]. The numbers of AP sites and methylated bases in MMS-treated and untreated cells were calculated based on the absorbance of this standard DNA.

### Measurement of cell viability

Log-phase or G_0_ cells were treated with 0–7.5 mM MMS at 37°C for 1 h; cells were then incubated in growth medium for 24 h. Cells were harvested and stained with PBS(-) (137 mM NaCl, 2.7 mM KCl, 4.3 mM Na_2_HPO_4_, 1.4 mM KH_2_PO_4_) containing 0.5% Trypan blue, and >500 cells were counted under a microscope. EC_50_ was determined by probit regression analysis using R [26].

### Real-time PCR

Total RNA was prepared with an RNeasy Plus Mini Kit (Qiagen, Hilden, Germany). cDNA was prepared via reverse transcription with 5 μg of total RNA and ReverTra Ace (Toyobo, Osaka, Japan) and an MJ Mini Personal thermal cycler (Bio-Rad). Real-time PCR was performed with primer sets shown in Table 1, SYBR Green Realtime PCR Master Mix (Toyobo), and a Line-gene Real-time PCR Detection System (Hangzhou Bioer Technology, Hangzhou, China).

**Table 1.** Primer sets used in this study

Mpg forward	5′-TGAATGTCTCTAGTCAAGGG-3
Mpg reverse	5′-AGTGCTTTTTCGGAGGGAGT-3′
Xrcc1 forward	5′-GGGAACTCGCCATACAGGAA-3′
Xrcc1 reverse	5′-GGCTCCACAGATGAGAACAC-3′
Polβ forward	5′-ACTGCCAGGAGTAGGAACAA-3′
Polβ reverse	5′-AGATGGTCCAATGCCAGTAA-3′
Apex1 forward	5′-TCTTGTGCCTCCAAGAGACC-3′
Apex1 reverse	5′-TGTTCTTCCTCGCCAATGCC-3′
β-Actin forward	5′-AGCGCAAGTACTCTGTGTGG-3′
β-Actin reverse	5′-AACGCAGCTCAGTAACAGTC-3′

### Immunoblotting

Cell lysates were prepared with SDS sample buffer (12.5 mM Tris-HCl, pH 6.8, 4% SDS, 10% sucrose, 10% 2-mercaptoethanol). The concentration of protein in each cell extract was measured with a Bio-Rad Protein assay (Bio-Rad). After SDS-PAGE, proteins were transferred to Immobilon-P (Merck KGaA, Darmstadt, Germany); membranes were then blocked, with 5% skim milk at 4°C for 16 h. Separated proteins were probed with primary antibodies, and antibody-bound antigens were detected with the appropriate secondary antibodies, ECL Western Blotting Detection Reagents (GE Healthcare, Little Chalfont, UK), and LAS-3000 (Fujifilm, Tokyo, Japan).

## RESULTS

### Sensitivities to MMS of log-phase and G_0_ cells

**Fig. 1.**
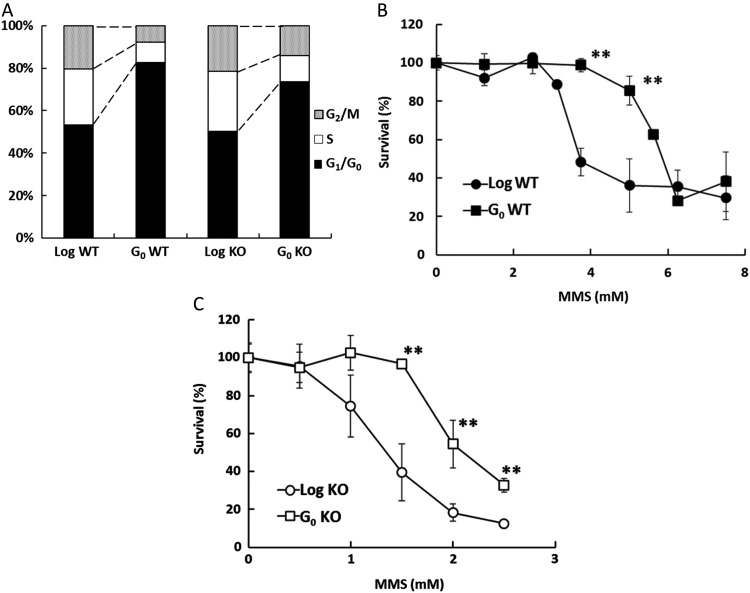
Sensitivities of log-phase and G_0_ cells to MMS. (**A**) Flow cytometry analysis of cell cycle phases in cultures of log-phase (Log) and G_0_ cells. For WT and *Pol β–*KO cells (KO), >80% and >70%, respectively, of the cells in the G_0_ cultures were synchronized in G_0_/G_1_ phase. (**B, C**) Cell survival of WT (B) and *Pol β–*KO cells (C) in log-phase or G_0_ after 1 h treatment with MMS. The cells were stained with 0.5% Trypan blue 24 h after removal of MMS; >500 cells were scored for viability under a microscope. **P < 0.01 for WT vs *Pol β–*KO cells. Data are represented as the mean of three experiments, and error bars indicate SD.

First, we examined whether growth-arrested (G_0_) MEFs were resistant to MMS, as observed in differentiated human neural cells [4]. G_0_ cells were prepared by serum starvation for 24 h. Based on cytometry analysis, >80% and >70% of WT and *Pol β–*KO cells, respectively were shown to be in a G_0_/G_1_ phase (Fig. 1A). To assess cell viability, log-phase and G_0_ cells were treated with 0–7.5 mM MMS and stained with 0.5% Trypan blue 24 h after treatment. At 3.75 or 5 mM MMS, G_0_ WT cells were more resistant to MMS than were log-phase WT cells (Fig. 1B); EC_50_ values were 4.22 and 5.84 mM for log-phase and G_0_ WT cells, respectively. Among *Pol β–*KO cells, there were also significant differences between log-phase and G_0_ cells at MMS concentrations from 1.5 to 2.5 mM (Fig. 1C); the respective EC_50_ values were 1.41 and 2.20 mM. As with results for differentiated cells [4], these findings indicated that non-proliferating cells were less sensitive to MMS than were proliferating cells, regardless of the presence or absence of POL β. The increased sensitivities to MMS resulting from the deficiency of POL β in log-phase and G_0_ cells (3.0 and 2.7-fold increases, respectively) indicated that POL β contributed to cell viability in both log-phase and G_0_ cells.

### BER activities in log-phase and G_0_ cells

Findings from cell viability assays revealed that G_0_ cells, either WT or *Pol β–*KO, were resistant to MMS. In G_0_ cells, MMS may have difficulty reacting with DNA bases because chromatin may be more tightly condensed. Thus, there may be fewer bases with MMS-induced damage in G_0_ cells than in log-phase cells. DNA damage after MMS treatment was measured via ARP assays. The numbers of AP sites and methylated bases induced by 1-mM MMS are shown in Table 2. In G_0_ cells, 1-mM MMS induced 0.27 (WT) and 0.30 (*Pol β–*KO) AP sites and 6.68 (WT) and 6.53 (*Pol β–*KO) methylated bases. In contrast, in log-phase, 1 mM MMS induced 1.06 (WT) and 0.61 (*Pol β–*KO) AP sites and 6.13 (WT) and 6.70 (*Pol β–*KO) methylated bases in the respective cell types. These results indicated that MPG formed significantly fewer AP sites in G_0_ than log-phase cells (*P* < 0.01); however, numbers of MMS-induced methylated bases did not differ significantly between G_0_ and log-phase cells. After 24 h of recovery from MMS treatment, more than 90% of methylated bases were repaired in log-phase cells of either genotype, WT or *Pol β–*KO. By contrast, only 70% and 61% of methylated bases were repaired in G_0_ cells (WT and *Pol β–*KO, respectively). These data indicated that both WT and *Pol β–*KO cells in G_0_ had less efficient repair of methylated bases than log-phase cells, but that they are more resistant to MMS than genotype-matched log-phase cells. It is conceivable that accumulation of AP sites in log-phase *Pol β–*KO cells may occur, because SP-BER does not proceed after MPG and APEX1 activity. However, for log-phase cells, significantly fewer AP sites had formed in *Pol β–*KO cells than in WT cells at 0 h; additionally, significantly more methylated bases remained in the *Pol β–*KO cells at 24 h (*P* < 0.05, Table 2). Thus, BER might be suppressed in log-phase *Pol β–*KO cells.

**Table 2.** Formation of AP sites and methylated bases in log-phase and G_0_ phase with 1-mM MMS treatment (/10^4^ nucleotides)

		AP sites		Methylated bases	
		WT	*Pol β–*KO	WT	*Pol β–*KO
Log phase	0 h	1.06 ± 0.39	0.61 ± 0.29	6.13 ± 1.02	6.70 ± 1.39
	24 h	0.062 ± 0.042	0.067 ± 0.009	0.29 ± 0.07	0.63 ± 0.18*
G_0_ phase	0 h	0.27 ± 0.04**	0.30 ± 0.01**	6.68 ± 0.36	6.53 ± 0.67
	24 h	0.10 ± 0.030	0.06 ± 0.016	2.04 ± 0.19**	2.56 ± 1.17**

*P < 0.05 vs WT. **P < 0.01 vs Log.

### Messenger RNA expression of Mpg and BER-related proteins in log-phase and G_0_ phase

**Fig. 2.**
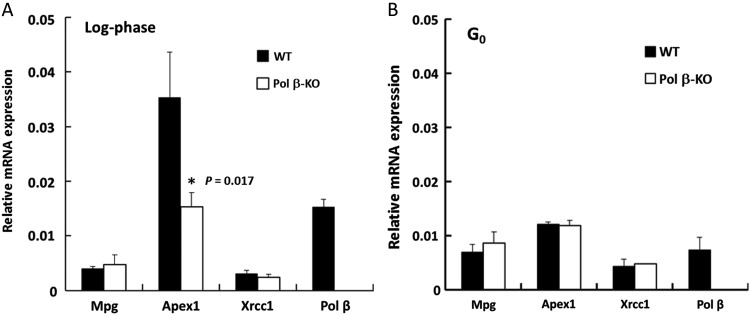
Levels of mRNA from MPG-initiated BER-related genes. mRNA levels for *Mpg* and related proteins in WT and *Pol β–*KO cells (KO) in log-phase (Log, **A**) and G_0_ (**B**) were measured via real-time PCR. Each expression level was normalized to that of β-Actin. *P < 0.05 for WT vs *Pol β*–KO cells. Data are represented as the mean of greater than three experiments, and error bars indicate SD.

We next examined the relationship between the low levels of AP sites in G_0_ cells and the amounts of *MPG* gene-products and related proteins. We had found that fewer AP sites formed in G_0_ cells than in log-phase cells during MMS treatment; additionally, MMS-induced methylated bases are mainly removed by MPG [27]. MPG activity is stimulated by proteins such as APEX1 and XRCC1 that work late in BER [28, 29]. Therefore, to determine the steady-state levels of repair proteins, the expression levels of mRNAs participating in MPG-initiated BER were measured in log-phase and G_0_ cells (Fig. 2).

Log-phase WT and *Pol β–*KO cells differed significantly with regard to *Apex1* mRNA levels (Fig. 2A). The amount of *Apex1* mRNA in log-phase *Pol β–*KO cells was 43% of that in log-phase WT cells, although both cell types expressed *Apex1* mRNA at similar levels in G_0_ cells (Fig. 2B). Expression levels of *Apex1* and *Pol β* mRNAs in G_0_ WT cells were 34% and 48%, respectively, of those in log-phase WT cells (Fig. 2A and B). In contrast, *Mpg* and *Xrcc1* mRNA levels did not differ significantly between WT and *Pol β–*KO cells for either log-phase or G_0_ cells (Fig. 2). These results indicated that expression of *Apex1* and *Pol β* mRNAs depended on cell proliferation. *Apex1* mRNA expression was also significantly suppressed in log-phase *Pol β–*KO cells, but it was not clear whether this difference was the result of the POL β deficiency (Fig. 2).

### Expression of MPG and BER-related proteins in log-phase and G_0_ phase

**Fig. 3.**
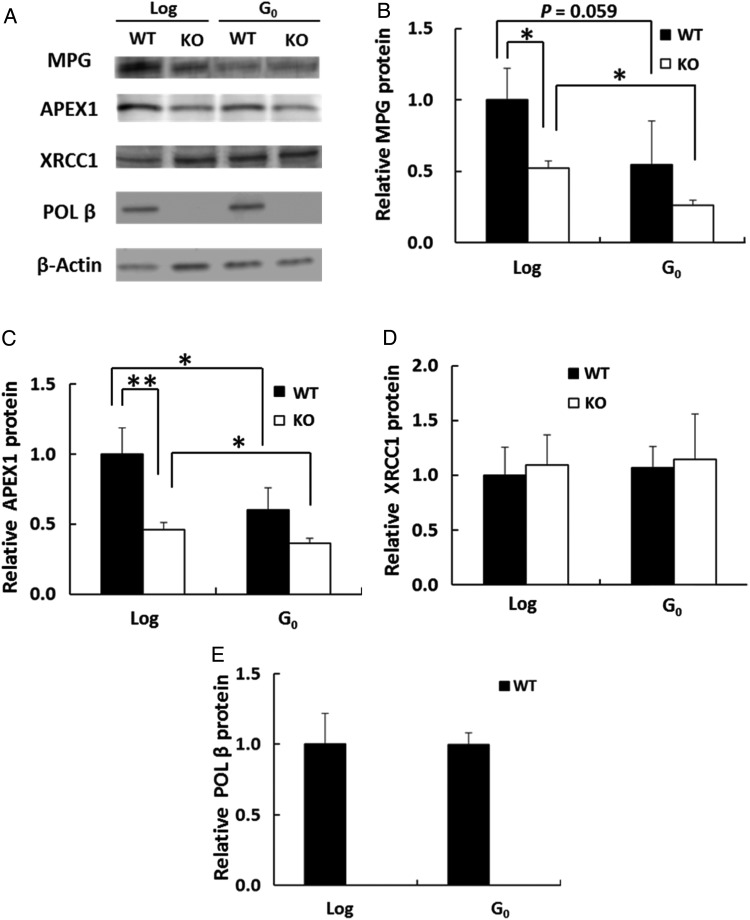
Amounts of MPG and MPG-related proteins in log-phase and G_0_ cells. The amount of each protein in WT and *Pol β*–KO cells (KO) in log-phase (Log) and G_0_ were measured by immunoblotting. (**A**) Proteins in cell lysate were separated by SDS-PAGE, and proteins were detected via antibody probes. (**B–E**) Quantification of immunoblot signals as shown in (A); each signal was normalized relative to the β-Actin signal. The amount in log-phase WT cells is shown as 1. *P < 0.05, **P < 0.01. Data are represented as the mean of three experiments, and error bars indicate SD.

To determine whether the findings for mRNA expression were reflected in protein levels, the amount of each protein in log-phase and G_0_ cells was examined on immunoblots (Fig. 3). Consistent with the amounts of mRNA expression, the amount of APEX1 protein in log-phase *Pol β–*KO cells was 48% of that in log-phase WT cells (Fig. 3C). For WT and *Pol β–*KO cells, APEX1 levels were significantly lower in G_0_ cells than in the genotype-matched log-phase cells (60% and 78%, respectively, Fig. 3C). Reduced MPG levels were also observed in G_0_ WT and G_0_ *Pol β–*KO cells (55% and 50% of the genotype-matched log-phase cells, respectively; Fig. 3B), suggesting that MPG had a more important role in growing cells than in quiescent cells and that the amount of MPG was regulated depending on cell proliferation state. Unexpectedly, the MPG protein level in log-phase *Pol β–*KO cells was 52% of that in log-phase WT cells, although *Mpg* mRNA levels did not differ between these cell types (Figs 2, 3B). In contrast, the POL β protein levels did not differ between G_0_ WT cells and log-phase WT cells, even though *Pol β* mRNA levels were lower in G_0_ WT cells (Figs 2, 3E). The XRCC1 protein level was constant regardless of POL β or the cell proliferation status (Fig. 3D). These results indicated that low levels of MPG and APEX1 proteins in G_0_ WT, G_0_ *Pol β–*KO, and log-phase *Pol β–*KO cells were responsible for the low number of AP sites and the slow removal of methylated bases in each of these cells types.

### Expression of PCNA protein in log-phase and G_0_ phase

**Fig. 4.**
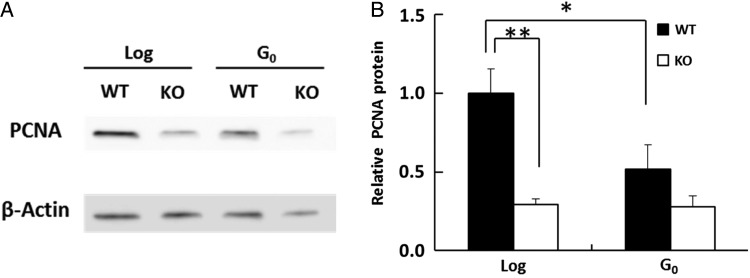
Amount of PCNA protein in log-phase and G_0_ cells. (**A**) Proteins in cell lysate from WT or *Pol β*–KO cells (KO) were separated by SDS-PAGE, and PCNA protein was detected via antibody staining. (**B**) Quantification of immunoblot signals as shown in (A); PCNA signals were normalized relative β-Actin signals. The amount in log-phase (Log) WT cells is shown as 1. **P < 0.01. Data are represented as the mean of greater than three experiments, and error bars indicate SD.

As shown in Fig. 3, MPG and APEX1 protein levels were lower in G_0_ WT, G_0_ *Pol β–*KO, and log-phase *Pol β–*KO cells than in log-phase WT cells, suggesting that SN-BER was not efficient in each of these three cell types. In log-phase *Pol β–*KO cells, SN-BER is unlikely to occur because POL β is also absent and because of low levels of MPG and APEX1. To investigate whether LP-BER, the other BER pathway, was affected in these cells, the amount of PCNA protein was measured. In log-phase *Pol β–*KO cells, the amount of PCNA was 29% of that in log-phase WT cells (Fig. 4A and B), whereas there was no significant difference in PCNA between G_0_ WT and G_0_ *Pol β–*KO cells. The PCNA protein level in G_0_ WT cells was 50% of that in log-phase WT cells, but there was no statistically significant growth state–dependent change in PCNA in *Pol β–*KO cells (Fig. 4B). These results suggested that LP-BER was also less efficient in G_0_ WT, G_0_ *Pol β–*KO, and log-phase *Pol β–*KO cells than in log-phase WT cells and that POL β deficiency may affect PCNA expression, especially in proliferating cells.

## DISCUSSION

MPG has a very prominent role in repair of spontaneously methylated or deaminated purines. The sensitivity to the MMS and DNA repair capacity of human neural cells depends on the level of cellular differentiation [4]; specifically, levels of SN-BER–related (APEX1, DNA Ligase III and XRCC1) and LP-BER–related enzymes are significantly lower in differentiated cells. MPG also interacts with PCNA and transcription activation factor estrogen receptor α [30, 31], and thus has a role in ensuring genomic integrity, mainly in proliferating cells. In this study, we examined the influence of POL β deficiency on MPG-initiated BER in proliferating and non-proliferating cells.

In good agreement with the previous findings [4], MMS resistance in G_0_ cells occurred regardless of the presence or absence of POL β (Fig. 1). The amount of MMS-induced methylated bases in G_0_ cells was similar to that in log-phase cells, but the number of AP sites in G_0_ cells was 26–49% of that in log-phase cells at 0 h; additionally, more methylated bases remained in G_0_ cells 24 h after removal of MMS (Table 2). The main MMS product, 7meG, is less cytotoxic than the AP sites formed from 7meG by MPG. It is conceivable that MMS resistance in G_0_ cells resulted from a reduction in the number of AP sites. Thus, we concluded that the excision activity of MPG was higher in log-phase cells than in G_0_ cells (Table 2) and consequently that MPG-initiated BER is more active in log-phase cells than in G_0_ cells.

Immunoblot-based measurements of expression of proteins related to MPG-initiated BER showed that the amounts of MPG, APEX1 and PCNA were each affected by cell proliferative state and POL β deficiency (Fig. 3B and C and Fig. 4). BER proteins are regulated by protein–protein interactions [32]. Because MPG activity is stimulated by APEX1 and PCNA [29, 30], decreased levels of MPG and these proteins may explain the low levels of BER activity in G_0_ cells.

Log-phase *Pol β–*KO cells, like G_0_ WT and *Pol β–*KO cells, had low levels of BER-related proteins and suppressed BER. Thus, the lack of POL β–dependent polymerase activity and/or dRP-lyase activity may have been primarily responsible for the hypersensitivity of *Pol β–*KO MEFs to MMS, presumably because these cells may not have been able to properly process BER intermediates.

The relative amounts of some proteins were not consistent with the relative amounts of the respective mRNA (Figs 2 and 3). Levels of APEX1 and POL β proteins are regulated by ubiquitination [33], which may explain the inconsistencies between mRNA and protein levels. Because an inconsistency was also observed for MPG protein and mRNA, it is likely that MPG is regulated in a similar way. We could not determine here whether the reduced levels of these proteins simply resulted from POL β deficiency. It is also conceivable that a clone with suppressed levels of BER enzymes was selected in the absence of POL β because the *Pol β–*KO cells retaining normal levels of MPG and APEX1 activity would be continuously threatened by accumulation of harmful BER intermediates.

## FUNDING

This work was supported in part by the Japan Society for the Promotion of Science (JSPS) Grant-in-Aid for Scientific Research (KAKENHI) Grant No. 25340036. Funding to pay the Open Access publication charges for this article was provided by Osaka Prefecture University.
